# Evaluation of serum CXC chemokine ligand 16 (CXCL16) as a novel inflammatory biomarker or familial Mediterranean fever disease

**DOI:** 10.3906/sag-2010-64

**Published:** 2021-04-30

**Authors:** Taner AKYOL, Tolga DÜZENLİ, Alpaslan TANOĞLU

**Affiliations:** 1 Department of Gastroenterology, Liv Hospital, Samsun Turkey; 2 Department of Gastroenterology, Hitit University Erol Olçok Training and Research Hospital, Çorum Turkey; 3 Department of Gastroenterology, Sultan Abdulhamid Han Training and Research Hospital, İstanbul Turkey

**Keywords:** Familial mediterranean fever, cxcl 16, inflammation, serumcxc chemokine ligand 16, biomarker

## Abstract

**Background/aim:**

Familial Mediterranean fever **(** FMF) is a disease that is mainly diagnosed with clinical features. Several well-known inflammatory markers increase in FMF. However, there is still a need for diagnostic tests for specifying FMF and monitoring inflammatory activity. CXCL16 is a chemokine produced by inflammatory cells that demonstrate efficacy in the acute phase response. In this study, we aimed to investigate the relationship between CXCL16 levels and FMF disease and to evaluate CXCL16 levels as a novel biomarker for FMF.

**Materials and methods:**

Fifty-three male patients diagnosed with FMF and sixty healthy individuals were included in this cross-sectional study. Blood samples were taken in the first 24 h of the attack periods. Serum soluble CXCL16 was evaluated by enzyme-linked immunosorbent assay (ELISA) method.

**Results:**

CXCL16 (P < 0.001), erythrocyte sedimentation rate (P < 0.001), C-reactive protein (P < 0.001), and fibrinogen (P = 0.005) were significantly higher in FMF group than in control group. Receiver operating characteristic (ROC) curve analysis revealed a cut off value of CXCL16 as 2.68 ng/ml with 83% sensitivity and 68% specificity (P < 0.001). Logistic regression analysis indicated that high CXCL16 and erythrocyte sedimentation rate levels were predictive parameters for FMF disease (OR 8.31; 95% CI 2.59-26.62; p <0.001) (OR 1.27; 95% CI 1.12-1.44; P < 0.001). There was no correlation between CXCL16 levels and attack frequency and disease duration (P = 0.395, P = 0.956).

**Conclusion:**

To the best of our knowledge, this is the first study evaluating serum soluble CXCL16 levels as a biomarker for FMF. CXCL16 levels were significantly higher and were predictive for monitoring inflammatory activity in patients with FMF. CXCL16 may be a promising biomarker for FMF diagnosis.

## 1. Introduction

Familial Mediterranean Fever (FMF) is a monogenic autoinflammatory disease characterized by recurrent fever and pain episodes mainly secondary to polyserositis, e.g., in the peritoneum, pleura, or joints. Although FMF has a distinct ethnic distribution, patients have been reported from many countries in the world. FMF is commonly detected in Sephardic Jews, Armenians, Turks, Greeks, Arabs, and Italians in the Mediterranean region [1,2]. FMF is a genetic disease resulting from MEFV gene variations [2,3]. This gene forms the protein of pyrin, which plays an important role in inflammation and apoptosis [2–4]. However, exact mechanism of pyrin pathway is not clear yet. One speculation for MEFV mutation is that pyrin and antiinflammatory protein production decreases and inflammatory conditions cannot be suppressed, thereby resulting in fever and inflammation in certain areas of the body.

There is currently no definitive diagnosis of FMF and it is diagnosed by clinical findings according to the Tel Hashomer criteria [1–5]. Ethnicity, family history, and the mutation in MEFV (Mediterranean fever) gene supports the diagnosis in patients with clinical findings although it is not a requirement. Other findings that contribute to the diagnosis are the high blood levels of acute phase reactants during attack periods of FMF. The major mechanism of pathogenesis for clinical manifestations includes overactivation of cytokine cascades [3,5]. Abnormal pyrin protein, which is proposed as a result of MEFV gene mutations, was presented to precipitate ineffective suppression of inflammation and was responsible for the inflammatory process [3].

Difficulties lead to delays in diagnosis of FMF. The main problems are atypical clinical presentations, which do not fully meet the diagnostic criteria and overlap diseases [6]. Considering that the average delay in diagnosis of FMF is 7.3 years; one can speculate that new, reliable, fast parameters are required for FMF diagnosis [7]. 

CXCL16 is a chemokine produced by inflammatory cells which are a member of the chemotactic cytokine superfamily and have an important role in inflammatory processes such as rheumatoid arthritis, inflammatory bowel disease, SLE, Behçet’s disease, and gout disease, as well as tissue damage and fibrosis [8–13]. CXCL16 activates CXC motif-receptor 6, which is synthesized in several cells, especially leukocytes [14]. Unlike the common chemokines, CXCL16 is expressed in two forms; on the surface as transmembrane protein and soluble CXCL16. sCXCL16 increases the migration of CXCR6 + cells (e.g. CD8 + T cells, CD4 + T cells, NK cells, plasma cells, monocytes) to secondary lymphoid organs and inflammation sites [15,16]. Thus, an inflammatory cascade is initiated via sCXCL16. 

In this study, our aim was to investigate the serum CXCL16 levels of patients in the FMF attack compared to healthy individuals and to investigate the role of CXCL16 on the diagnosis and activity of FMF disease.

## 2. Materials and methods

### 2.1. Study population

Fifty-three male patients diagnosed with FMF and sixty healthy age-matched male individuals were enrolled in this cross-sectional study. Local ethics committee approved the study. Informed consents were obtained from all participants.

The FMF attack period was diagnosed by Tel Hashomer criteria and combining high levels of acute phase reactants (APR) with positive physical examination. All patients had peritonitis in terms of phenotype characteristics. 

All findings of the patients were recorded for further evaluation and classification. Blood samples were obtained, and physical examination was performed to the patients who referred with FMF attack.

We performed a strict exclusion criteria. Patients using nonsteroidal antiinflammatory drugs, steroids, immunosuppressants, or immune regulatory drugs, and patients with cardiovascular disease, acute or chronic liver, or kidney disease, chronic obstructive pulmonary disease, rheumatological disease, malignancy, thyroid disease, immune deficiency, hypertension, diabetes mellitus, acute/chronic infection, bleeding disease, and those who did not meet the study criteria were excluded from the study. Blood samples were obtained in the first 24 h of the attack period. 

### 2.2. CXCL16 measurements

Blood samples were centrifuged at 5000 rpm for 5 min and 2 cc serum was separated into eppendorf tubes for CXCL16 tests and stored at –80°C. After a sufficient sample size was reached, all samples were tested in the biochemistry laboratory. CXCL16 was analyzed by ELISA (Human CXCL 16 Immunoassay ELISA Kit, R&D Systems Inc Minneapolis, USA) according to the manufacturer’s recommendations.

### 2.3. Statistical analysis 

SPSS 20.0 (IBM Corp., Armonk, NY, USA) was used for statistical analysis. Descriptive statistics were performed and Kolmogorov–Smirnov test was used to check the data for normal distribution. Comparisons of the groups were made by student T test for the data with normal distribution, and Mann–Whitney U test for the data without normal distribution. Categorical data were assessed by Chi-square test. Receiver operating characteristic (ROC) analysis was used to evaluate the sensitivity and specificity of CXCL16 in FMF patients. Logistic regression analysis was performed to determine the parameters predicting FMF attack. Pearson and Spearman analyses were used for the correlation analysis. The P value <0.05 was considered statistically significant.

## 3. Results

Demographic and clinical data of patient groups are presented in Table 1. CXCL16 (P < 0.001), CRP (P < 0.001), fibrinogen (P = 0.005), and ESR (P < 0.001) were significantly higher in the FMF group compared to the control group, while BMI (P = 0.011) was higher in healthy individuals. 

**Table 1:** Demographic and clinical characteristics of patients with FMF and healthy controls.

Group	FMF patients (n = 53)	Healthy controls (n = 60)	P value
Age, years	24.1 ± 3.4	27.2 ± 6.7	0.090
BMI, kg/m2	23.0 ± 3.5	24.6 ± 2.8	0.011*
Smoking, n %	28/53. 52.8%	27/60. 45%	0.370
Disease duration, years	12.6 ± 5.9		
Attack frequency, per year	2.6 ± 0.9		
WBC, 10³/μL	7.291 ± 2.225	6.851 ± 1.400	0.236
CRP, mg/L	10.5 ± 11.1	5.1 ± 12.5	<0.001*
ESR, mm/h	19.2± 15.4	4.8 ± 3.4	<0.001*
Fibrinogen, mg/dL	298.6 ± 94.9	236.4 ± 52.2	0.005*
Glucose, mg/dL	82.9 ± 8.3	84.2 ± 8.1	0.412
LDL-Cholesterol, mg/dL	89 ± 29.3	94.9 ± 26.5	0.299
Triglyceride, mg/dL	115.1 ± 56.5	105.3 ± 57.9	0.239
CXCL 16, ng/mL	3.20 ± 0.62	2.57 ± 0.38	<0.001*

BMI body-mass index, WBC white blood cell,ESR erythrocyte sedimentation rate, CRP C reactive protein, CXCL16 serum CXC chemokine ligand 16.*P < 0.05 was considered statistically significant.

ROC curve analysis revealed a cut off value of CXCL16 as 2.68 ng/mL with 83% sensitivity and 68% specificity (P < 0.001) (Table 2, Figure). Logistic regression analysis indicated that high CXCL16 and sedimentation levels were predictive parameters for FMF disease (OR 8.31; 95% CI 2.59–26.62; P < 0.001) (OR 1.27; 95% CI 1.12–1.44; P < 0.001) (Table 3). There was no correlation between CXCL16 levels and annual attack frequency and disease duration of FMF patients (P = 0.395, P = 0.956).

**Table 2:** Receiver operating characteristic (ROC) analysis of CXCL16 in patients with FMF.

	AUC (95%)	Cut off value	p	Sensitivity (%)	Specificity (%)
CXCL16	0.820 (0.739–0.900)	2.68 ng/mL	<0.001*	83	68

* P < 0,05 was considered statistically significant.

**Table 3:** Logistic regression analysis of parameters associated with FMF.

Variable	Odds ratio	95 % confidence interval	P value
CXCL16 (> cut-off value 2,68 ng/mL)	8.31	2.59–26.62	<0.001*
ESR, mm/h	1.27	1.12–1.44	<0.001*

* P < 0,05 was considered statistically significant.

**Figure:**
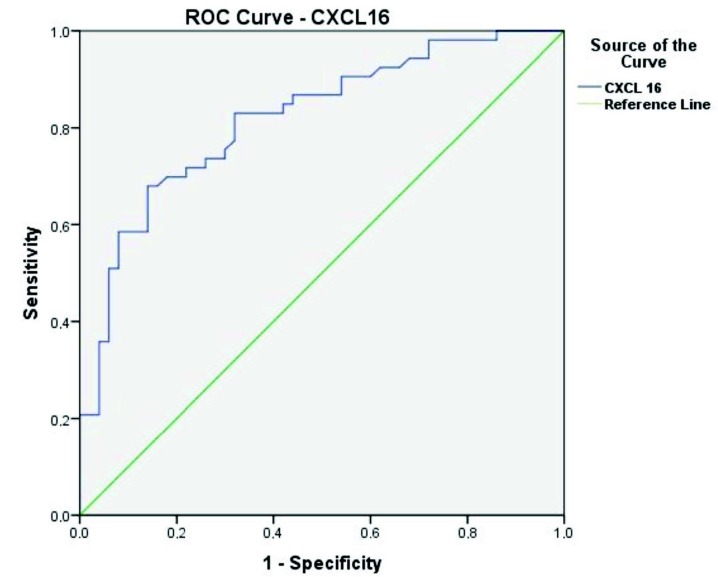
Reciever operating characteristic (ROC) curve of CXCL16 in patients with FMF.

## 4. Discussion

To the best of our knowledge, this is the first study evaluating serum soluble CXCL16 levels as a biomarker for FMF. In this study, we showed that patients with FMF attacks had higher serum CXCL16 levels compared to healthy individuals and CXCL16 had a predictive role in the diagnosis of FMF disease.

Higher ESR, CRP, fibrinogen, white blood cell count, and serum amyloid A (SAA) are expected results in FMF disease compared to the attack-free period [17,18]. However, in a systematic review investigating acute phase reactants used for FMF diagnosis, Erer et al. reported that there was no effective acute phase reactant to diagnose FMF disease [18]. 

Actually, maybe as one of the limitations in our study, we could not test the SAA between these parameters because SAA was not among our routine tests in our biochemistry lab. Nevertheless, Çakan et al. recently reported that SAA is a sensitive but nonspecific marker to show inflammation in FMF [19]. The authors demonstrated that SAA levels increase significantly in febrile upper respiratory infections [19]. 

Innate immune system in FMF patients is proposed to be disrupted and the disease progresses with episodes of systemic inflammation [20]. Innate immune system activates the adaptive immune system by antigen presenting cells. Thus, B and T cells respond and result as disease symptoms [20]. IL-1β, IL-1α, TNF α, TNF β and IL-6 play an important role in these mechanisms [5]. CXCL16 has been reported to play an activating role in the pathophysiology of these inflammatory cascades [14]. In addition, Funk T et al. noted that dendritic cells have critical roles in FMF pathogenesis and disease activation [21]. CXCL16 is also known to be expressed in dentritic cells and is effective in triggering inflammation [14]. Inflammatory cytokines, such as TNF α and interferon-gamma, which play an important role in the pathogenesis of FMF, have also been shown to increase CXCL16 production [22]. In the light of these mechanisms, our findings suggest that high CXCL16 levels can be a relevant diagnostic marker for FMF disease.

Literature states an increase of CXCL16 in several inflammatory diseases [8–13,16]. In our study, logistic regression analysis indicated that high CXCL16 and ESR levels were predictive parameters for FMF disease. We think that the major advantage of CXCL16 over common widely available conventional APRs would be its OR of 8.31 for predicting FMF while ESR’s OR was 1.27. In addition to that, ROC curve analysis revealed satisfactory scores of sensitivity and specificity for CXCL16. Eventually, there is need for further prospective studies to evaluate CXCL16 in different periods of FMF disease and its role at differential diagnosis.

As an interesting result of our study, there was not any correlation between CXCL16 levels and attack frequency and disease duration of FMF patients. This result may indicate that CXCL16 is effective especially in attack period of FMF pathogenesis. On the other hand, colchicine treatment can cause this result as it has antiinflammatory effects and can cause a decrease in proinflammatory cytokine levels [2–4]. Another reason may be the cross-sectional design of the study that the samples reflect only one time period. 

Another finding in our study was that BMI (body mass index) values were significantly higher in control group than in FMF patients. A relationship has been proposed between obesity and CXCL16 [23]. In this regard, Lopes et al. reported that the overweight increases CXCL16 levels in young adults (18–30 years old) [23]. We consider that this finding of BMI values, which were significantly lower in patients with FMF than healthy controls, may consolidate the diagnostic value of CXCL16 in patients with FMF.

There are other new candidate biomarkers for FMF in the literature [17,24–31]. Pentraxin-3, omentin, fetuin, calprotectin, serum amyloid A, CD144+, and CD146+ as circulating endothelial microparticles, endocan, chitotriosidase, serum matrix metalloproteinase-9 and tissue inhibitor of metalloproteinase-1, S10012A and resolvin D1 has been investigated and had promising results. However, there is still need for prospective large cohort studies to use these novel biomarkers in daily practice. This study has some limitations. First, the study population was small. Second, all study participants were male because the study was conducted at a tertiary military center. However, we anticipate that this limitation will not weaken our results due to the efficient homogenization between the groups. Third, CXCL was only determined during attack period and was not analyzed in between attacks. On the other hand, our study population is young and they have no comorbid situations. Thus our strict exclusion criteria render our research valuable.

In conclusion, we showed that CXCL16 levels were significantly higher in patients with FMF than in healthy individuals. Serum soluble CXCL16 levels may be a novel biomarker for FMF disease. Prospective, randomized, large studies are needed for elucidating the role of CXCL16 in the pathogenesis of FMF.

## Informed consent

All participants provided informed consent. The approval from the ethics committee of GATA Haydarpaşa Training Hospital (Sultan Abdulhamid Han Training and Research Hospital) was obtained for this study. The study was carried out in accordance the World Medical Association’s Declaration of Helsinki. 
